# Bifunctional Avidin with Covalently Modifiable Ligand Binding Site

**DOI:** 10.1371/journal.pone.0016576

**Published:** 2011-01-27

**Authors:** Jenni Leppiniemi, Juha A. E. Määttä, Henrik Hammaren, Mikko Soikkeli, Mikko Laitaoja, Janne Jänis, Markku S. Kulomaa, Vesa P. Hytönen

**Affiliations:** 1 Institute of Medical Technology, University of Tampere and Tampere University Hospital, Tampere, Finland; 2 Department of Chemistry, University of Eastern Finland, Joensuu, Finland; Cardiff University, United Kingdom

## Abstract

The extensive use of avidin and streptavidin in life sciences originates from the extraordinary tight biotin-binding affinity of these tetrameric proteins. Numerous studies have been performed to modify the biotin-binding affinity of (strept)avidin to improve the existing applications. Even so, (strept)avidin greatly favours its natural ligand, biotin. Here we engineered the biotin-binding pocket of avidin with a single point mutation S16C and thus introduced a chemically active thiol group, which could be covalently coupled with thiol-reactive molecules. This approach was applied to the previously reported bivalent dual chain avidin by modifying one binding site while preserving the other one intact. Maleimide was then coupled to the modified binding site resulting in a decrease in biotin affinity. Furthermore, we showed that this thiol could be covalently coupled to other maleimide derivatives, for instance fluorescent labels, allowing intratetrameric FRET. The bifunctional avidins described here provide improved and novel tools for applications such as the biofunctionalization of surfaces.

## Introduction

The extremely high affinity of avidin and streptavidin towards D-biotin (K_d_≈6×10^−16^ M for avidin and K_d_≈4×10^−14^ M for streptavidin) [1]–[3] is the reason why these proteins are widely applied in life sciences. Biotin occupies a structurally optimal cavity inside the eight-stranded beta barrel of (strept)avidin forming several hydrogen bonds with the protein [4]–[6]. Previous studies have indicated the importance of a particular set of residues in this interaction. However, removal of a single hydrogen-bonding residue weakened biotin binding to (strept)avidin only moderately [7]–[11]. Therefore, fairly radical mutations have to be applied in order to extinguish the biotin-binding activity of (strept)avidin as previously reviewed [2]. In another approach, biotin was chemically adjusted to increase its binding affinity, but with poor results [12]. Two studies reported rational engineering of streptavidin, where the ligand-binding specificity of mutant streptavidin was altered from biotin to its analog, 2-iminobiotin [13], or to biotin-4-fluorescein [14]. Avidin was also modified to improve the binding of 4′-hydroxyazobenzene-2-carboxylic acid (HABA) resulting in a change of several orders of magnitude in the binding affinity as compared to wild type (wt) avidin [15]. However, avidin could not be converted to favour binding of HABA over biotin. Overall, the site-directed modulation of the ligand-binding specificity of (strept)avidins towards ligands other than biotin or its derivatives appears to be challenging.

The independent control of individual binding sites within tetrameric (strept)avidin has been the motivation for numerous studies. A monovalent streptavidin tetramer with only one functional biotin binding site was reported [16]: The unmodified and modified monomers were combined in a denaturated state at a molar ratio of 1∶3 before refolding. This method created a mixture of tetramers of different compositions from monovalency to tetravalency following isolation of the monovalent protein from the oligovalent forms. This laborious procedure could be avoided by genetically joining two or four modified subunits together into a polypeptide chain like in the dual chain and single chain avidins previously reported [17], [18]. The same approach has since also been applied to streptavidin [14].

**Figure 1:**
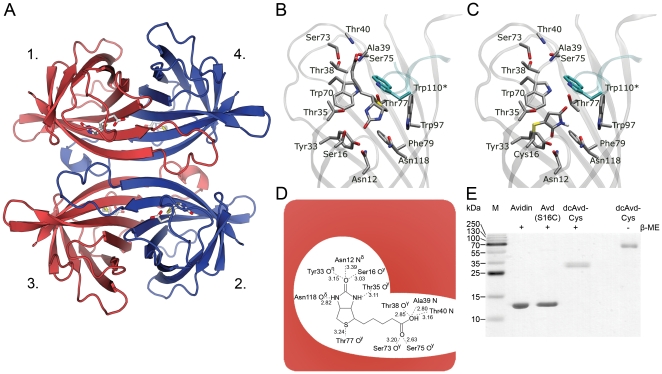
Avidin-ligand interaction. (**A**) Avidin tetramer (PDB 2AVI), with subunits numbered according to [5]. Subunits are coloured to red and blue according to dual chain avidin subunits; cpAvd5→4 are shown in red and cpAvd6→5 are shown in blue. (**B**) A cartoon model of wild type (wt) avidin (PDB 2AVI) showing the biotin-binding contact residues in sticks. Oxygen atoms are shown in red, nitrogens in blue, sulphurs in yellow, and carbon atoms in grey. The W110 residue from the neighboring subunit is highlighted in cyan. (**C**) The cartoon model of the maleimide-treated binding site of Avd(S16C) showing a thioether bond between Cys16 and maleimide. (**D**) A schematic of the hydrogen bonds between wt avidin and biotin. Bond lengths in ångströms are shown according to (PDB 2avi) [6]. (**E**) SDS-PAGE analysis of recombinant proteins shows high purity and correct molecular weights. In the presence (+) of the reducing agent β-mercaptoethanol (β-ME), Avidin, Avd(S16C) and dcAvd-Cys (more specifically dcAvd(I117C_5→4_S16C,V115H_6→5_)) exist as monomers of sizes ∼14.5 kDa, ∼14.5 kDa and ∼30 kDa, respectively. DcAvd-Cys without (-) the reducing agent exists mainly in the form of a disulfide linked dimer, ∼60 kDa. M, molecular weight marker.

Dual chain avidin (dcAvd) is a subunit fusion of two different circularly permuted avidin monomers, circularly permuted avidin 5→4 (cpAvd5→4) and circularly permuted avidin 6→5 (cpAvd6→5) [17]. Two dcAvd subunits, both with two binding sites, form a dimer with four binding sites (Figure 1A). The subunits can independently be modified, making it possible to create avidins with altered binding sites, for instance for low-affinity and high-affinity binding of a ligand within a single protein molecule [7]. The use of a disulphide bridge (I117C_5→4_, numbering according to wt avidin) combined with a critical mutation (V115H_6→5_) in the interface between subunits [19] made it possible to develop dcAvds with defined quaternary structures [20]. DcAvd provides a scaffold that can be modified not only to alternate affinity for a ligand, but also to change specificity – in other words to favour another ligand molecule instead of biotin.

In order to overcome the challenge of modifying the extremely high biotin-binding affinity of (strept)avidin, we established a novel concept, in which a cysteine residue was introduced into the ligand binding site of avidin. The thiol group of the cysteine could then be covalently modified leading to the inhibition of biotin-binding. In addition, this allowed the selective covalent linking of thiol group reactive molecules to avidin. Furthermore, an avidin with two specific ligand-binding sites was created by applying the same modification to dcAvd.

## Results

### Serine 16 replaced by cysteine can be modified by maleimide

Serine 16 is positioned in the biotin-binding pocket of avidin and is hydrogen bonded to a ureido oxygen in biotin in the avidin-biotin complex (PDB 2AVI; Figure 1B,D). Our strategy was to replace serine 16 with a cysteine to introduce a thiol group to the polypeptide available for covalent bonding with small thiol-reactive molecules. Importantly, being located inside the binding pocket, the free thiol group is unable to form disulphide bond between another avidin subunit, which would lead to oligomeric assemblies [21], and therefore reducing conditions were not needed for protein handling. Cysteine 16 could then be modified by maleimide, which reacts specifically with thiol group in chemically mild conditions forming a stable thioether linkage (Figure 1C). Furthermore, Cys16 could be covalently coupled to other maleimide derivatives, as demonstrated here by linking a maleimide-activated fluorescent probe to dcAvd(I117C_5→4_S16C,V115H_6→5_), referred to as dcAvd-Cys from now on. DcAvd-Cys could be coupled to two different kinds of molecules, to thiol-reactive molecules as well as to biotinylated molecules.

We produced avidin and Avd(S16C) in *E. coli* cells and dcAvd-Cys in baculovirus infected Sf9 insect cells. This was followed by successful protein purification by one-step 2-iminobiotin affinity chromatography [22] indicating protein solubility and activity in the form of biotin binding. The purified proteins were of the correct size according to SDS-PAGE analysis (Figure 1E). Non-reducing SDS-PAGE showed the formation of a thioether between the dcAvd-Cys subunits (Figure 1E).

**Figure 2:**
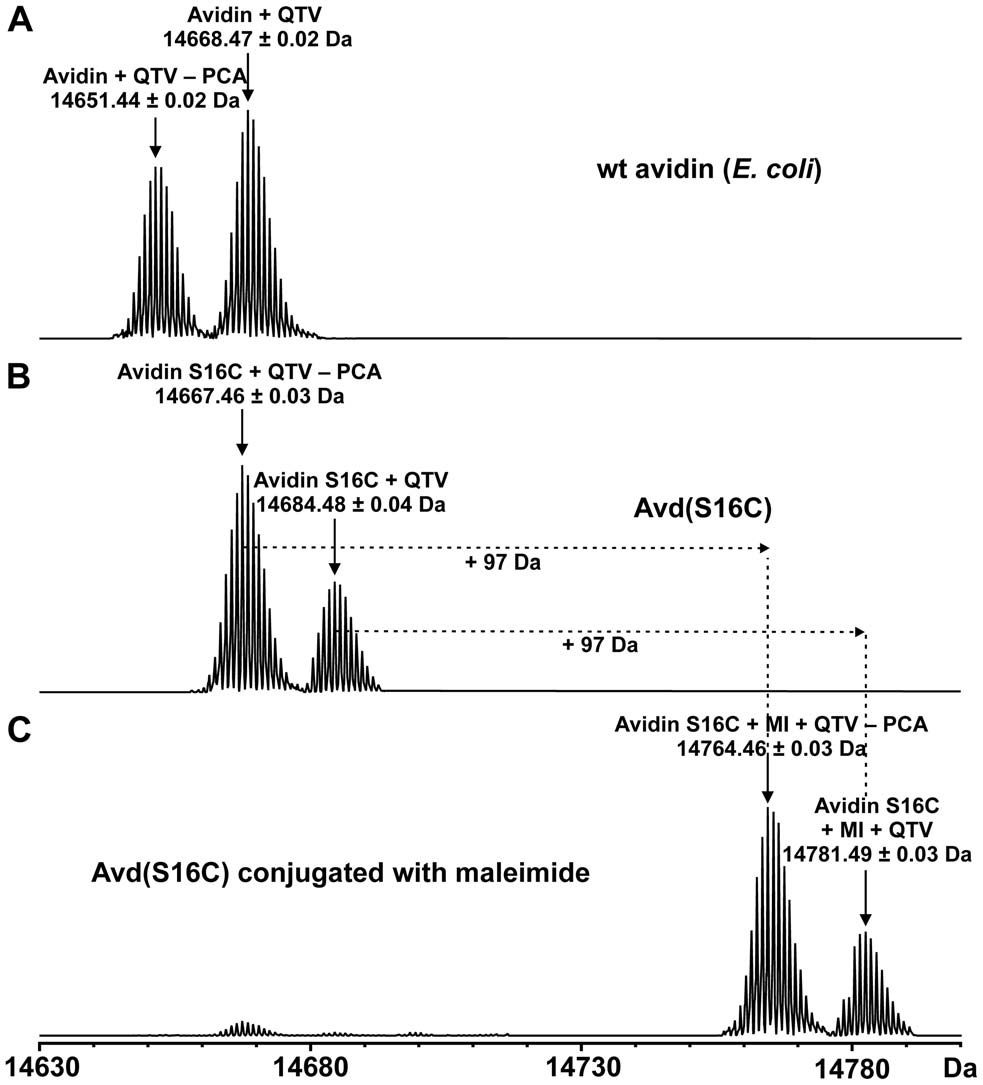
A charge-deconvoluted ESI FT-ICR mass spectra for avidin, Avd(S16C) and Avd(S16C) conjugated with maleimide. The mass spectra showed that both avidin (**A**) and Avd(S16C) (**B**) have three additional N-terminal amino acid residues (QTV) of the *B. avium* OmpA signal peptide and they both contain an intramolecular disulfide bridge (theoretical masses are 14668.43 and 14684.41 Da for avidin and Avd(S16C), respectively). The proteins appeared in two different forms separated by 17 Da due to the partial cyclization of an N-terminal glutamine residue into the pyrrolidone carboxylic acid (PCA) form. The mass spectrum of Avd(S16C) conjugated with maleimide (MI) (**C**) showed a mass increase of +97 Da, indicating a formation of a covalent thioether linkage with Cys16 (theoretical mass 14781.43 Da). The small amount of unreacted protein present in the sample indicated high coupling efficiency between maleimide and the cysteine-residue.

We used high-resolution electrospray ionization Fourier transform ion cyclotron resonance (ESI FT-ICR) mass spectrometry to confirm protein primary structures and to detect the efficiency of coupling maleimide into Avd(S16C). Figure 2 shows isotopically-resolved, charge-deconvoluted ESI FT-ICR mass spectra for wt avidin, Avd(S16C) and Avd(S16C) coupled with maleimide (MI). Based on the mass data, both wt avidin and Avd(S16C) had three additional amino acid residues (QTV) of the *B. avium* OmpA signal peptide in the N-terminus of the protein and contained an intramolecular disulfide bridge, Cys4–Cys83 (the theoretical masses are 14668.43 and 14684.41 Da for wt avidin and Avd(S16C), respectively). In addition, both proteins appeared in two different forms, separated by 17 Da, due to the partial cyclization of an N-terminal glutamine residue into the pyrrolidone carboxylic acid (PCA) form (Figure 2A,B). No protein dimers or higher oligomers were detected, indicating the absence of C16-mediated tetramer crosslinking. We incubated a high molar excess of maleimide with Avd(S16C) to measure coupling efficiency with the free cysteine present in the protein structure. A mass spectrum of Avd(S16C) treated with maleimide (Figure 2C) consistently showed a mass increase of +97 Da, indicating the formation of a covalent thioether linkage, presumably with Cys16 (theoretical mass 14781.43 Da). The coupling efficiency appeared to be high since only a small amount of unreacted protein was present in the sample. We detected no other protein modifications upon the maleimide treatment. We obtained similar results when Avd(S16C) was treated with N-ethylmaleimide, another thiol-reactive maleimide derivative, showing nearly 100% coupling efficiency (data not presented). In contrast, treatment of wt avidin with maleimide or N-ethylmaleimide showed no reaction at all, as expected, indicating high specificity of the two reagents towards free thiol groups in these conditions.

To further verify the presence of the maleimide-coupled residue, wt avidin as well as Avd(S16C), with or without maleimide treatment, were digested in solution with trypsin (data presented as Table S1 in Supporting Information). The full sequence coverage was obtained, except in the case of Avd(S16C) (87%), represented by up to 20 specific tryptic peptides. Among the identified peptides for Avd(S16C) treated with maleimide, a peptide having a monoisotopic mass of 1947.8630 Da was observed, consistent with the maleimide conjugation into residues 10–26 (theoretical mass 1947.8567 Da). With untreated Avd(S16C), this peptide was absent, but a peptide having a monoisotopic mass of 1850.8512 Da was observed instead, corresponding to the uncoupled tryptic peptide 10–26. These results indicate that maleimide was effectively and specifically conjugated to the Cys16 residue in the modified Avd(S16C). The digestion data also confirmed the presence of intramolecular disulfide bridge (disulfide linked tryptic peptides 3–9+72–94 and 4–9+72–94) as well as the N-terminal PCA modification.

### The S16C mutation does not prevent biotin binding but decreases affinity to biotin

The thermal stability of Avd(S16C) in the absence of biotin was comparable to that of wt avidin (Table 1, see also Figure S1). However, the S16C mutation had a negative effect on the thermal stability of the quaternary structure of wt avidin in the presence of biotin, since the thermal transition temperature decreased about 20°C. This was expected, as the mutation was targeted to one of the key residues participating in ligand binding. Similarly to dcAvd(I117C_5→4,_V115H_6→5_), dcAvd-Cys was a monomer at room temperature (RT, 23±1°C) in the absence of biotin. The presence of biotin stabilized dcAvd-Cys, although it lost its pseudotetrameric structure at 60°C, which is about 20°C lower than the thermal transition temperature determined for dcAvd(I117C_5→4,_V115H_6→5_) [20].

**Figure 3:**
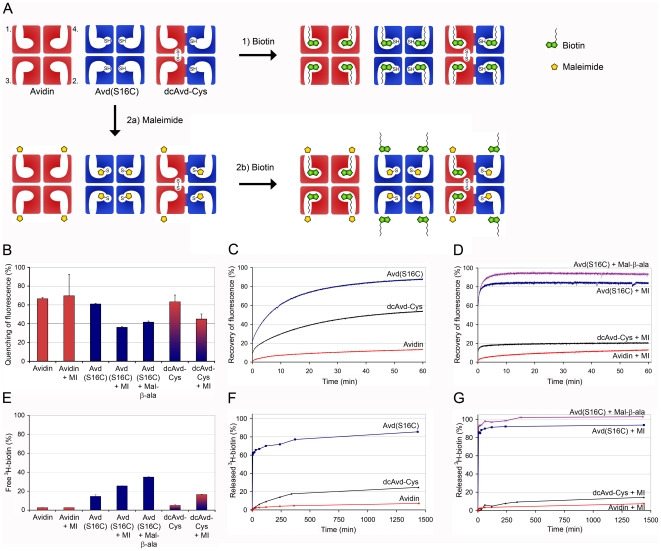
Biotin dissociation asays. (**A**) A schematic presentation of the four binding sites in avidin, Avd(S16C) and dcAvd(I117C_5→4_S16C,V115H_6→5_) (dcAvd-Cys). Biotin could bind to both of the S16C-mutated and unmodified binding sites (scheme 1). The biotin binding to Avd(S16C) and dcAvd-Cys could be inhibited with maleimide, which forms a covalent bond with a thiol group in the modified binding site (scheme 2 a,b). (**B**) The level of quenching of fluorescence of BF560-biotin after it was bound to protein indicated different biotin-binding abilities for different protein forms with or without chemical coupling to maleimide (MI) or to N-maleoyl-β-alanine (Mal-β-ala). The means for duplicate measurements are shown ± s.d. (**C**, **D**) The recovery of fluorescence of BF560-biotin after the addition of a 100-fold excess of free biotin indicates dissociation of the BF560-biotin-protein complex. (**E**) Proteins were incubated with ^3^H-biotin and unbound ^3^H-biotin was filtered and measured with a scintillation counter. Means of triplicate measurements are shown ± s.d. (**F, G**) The release of ^3^H-biotin after addition of cold biotin measured at different time points.

As was stated above, Avd(S16C) efficiently bound to a 2-iminobiotin affinity matrix, which qualitatively suggested relatively high biotin-binding affinity. We analyzed the ligand binding properties of the modified avidins with a fluorescent biotin conjugate, BF560-biotin, by measuring the quenching of fluorescence after protein binding at RT. The quenching of fluorescence (Figure 3B) of wt avidin, Avd(S16C) and dcAvd-Cys were fairly equal, 66.5±1.3, 61.0±0.6 and 63.2±7.3% respectively, indicating that the S16C mutation had only a modest effect on the binding of the fluorescent biotin. However, after addition of free biotin, the recovery of fluorescence (Figure 3C) of Avd(S16C) was over 15-fold faster (k_diss_ = 3.7×10^−4^ s^−1^) compared to wt avidin (k_diss_ = 2.4×10^−5^ s^−1^), indicating an increase in the dissociation rate and thus decreased affinity to the conjugated biotin. After one hour the recovery of fluorescence was nearly 15% with avidin and 85% with Avd(S16C). When compared to that of the previously analyzed Avd(S16A) with its dissociation rate constant of 3.6×10^−4^ s^−1^ and fluorescence recovery of 74.5% after one hour [7], we can conclude that the mutations S16A and S16C affect the biotin-binding properties of avidin in a similar fashion. The recovery of fluorescence for dcAvd-Cys was slightly over 50% after one hour and the dissociation rate constant 1.3×10^−4^ s^−1^, being thus five-fold faster than for wt avidin but three-fold slower than for Avd(S16C). Because the pseudotetrameric dcAvd-Cys has two S16C-modified and two unmodified binding sites, the result appears logical: dcAvd-Cys behaves like an independent combination of wt avidin and Avd(S16C).

We used ^3^H-biotin to confirm the results we obtained for ligand binding with fluorescently labelled biotin. Incubation of ^3^H-biotin with proteins at RT following filtering and scintillation counting of ^3^H-biotin indicated differences in biotin-binding abilities. The results appeared logical, since wt avidin bound almost all of the ^3^H-biotin and the amount of free ^3^H-biotin was 2.7±0.1% (Figure 3E). The ^3^H-biotin-binding ability of Avd(S16C) was somewhat reduced, since the amount of free biotin was 14.6±1.6%. The amount of unbound ^3^H-biotin in the case of dcAvd-Cys was 5.1±0.6% indicating a slightly reduced ability to bind ^3^H-biotin as compared to wt avidin. The release of the bound ^3^H-biotin was measured after the addition a 1000-fold excess of cold biotin and the dissociation from Avd(S16C) (k_diss_ = 6.4×10^−4^ s^−1^) approached 85% after 24 hours, and therefore, dissociation was increased nearly 18-fold as compared to wt avidin, which had released only 7% of the bound ^3^H-biotin after 24 hours (k_diss_ = 3.6×10^−5^ s^−1^) (Figure 3F). At 24 hours after addition of cold biotin the dissociation of the bound ^3^H-biotin from dcAvd-Cys approached 25% and was nearly five-fold faster (k_diss_ = 1.7×10^−4^ s^−1^) than from wt avidin.

According to both assays biotin dissociation was much faster from Avd(S16C) as compared to wt avidin. However, for dcAvd-Cys the results of the two assays differed slightly. A possible reason for this could be the structural differences of the used ligands. The fluorescent label itself is large compared to biotin and when attached to biotin via a linker, the label could affect biotin-binding properties. D-[8,9-^3^H]-biotin with two tritiums, on the other hand, is structurally almost identical with natural D-biotin.

### Blocking the biotin binding of avidins by coupling with maleimide

We coupled maleimide to the binding sites of the modified avidins (Figure 3A) and tested whether this had an effect on the biotin binding-affinity as determined by using fluorescently labelled BF560-biotin. Maleimide had no effect on the binding properties of wt avidin (Figure 3B). In contrast, maleimide coupled to Avd(S16C) disrupted the binding of fluorescent biotin, which was seen as a decrease in the quenching of fluorescence to 36.1±0.7%. The recovery of fluorescence after the addition of free biotin was rapid and most of the complexes were dissociated already after one minute from the beginning of the measurement (Figure 3D), indicating significant decrease in the affinity for biotin. Ser16 is located in the loop area of β-barrel of avidin and therefore it is possible that the loop may partly distort so that biotin may fit in the binding site, although maleimide is already covalently coupled to modified Cys16. Therefore, we were interested to know whether maleimide with an attached side group would prevent the binding of biotin to the modified site more efficiently. We selected N-maleoyl-β-alanine (Mal-β-ala), a maleimide with an attached three-carbon chain, and coupled it to Avd(S16C). However, the quenching of fluorescence did not decrease, although the recovery of fluorescence after the addition of free biotin was even faster in the case of Mal-β-ala than bare maleimide (Figure 3D) indicating that larger molecules can decrease the affinity of Avd(S16C) for biotin more efficiently.

The quenching of fluorescence of dcAvd-Cys coupled with maleimide was 45.0±5.3% (Figure 3B), which was an intermediate of the values measured for wt avidin and Avd(S16C). Therefore, it seems that maleimide was able to inhibit the binding of biotin to the cysteine-modified binding site in the cpAvd6→5 domain of the dcAvd-Cys. As a consequence, the remaining biotin-binding activity of dcAvd-Cys would depend mainly on the cpAvd5→4 domain, which closely resembles that of wt avidin [17], [20]. The rapid burst (∼10%) in the recovery of fluorescence just after the addition of free biotin was most probably mainly associated with the dissociation of fluorescent biotin from the cpAvd6→5 domain with the S16C modified binding site. The burst is followed by a slow phase of biotin dissociation from the cpAvd5→4 domain with an unmodified ligand-binding site. After an hour the recovery of fluorescence of dcAvd-Cys-MI was 26.3±7.8% suggesting that the cpAvd5→4 domain had a wt-like biotin-binding affinity.

Again, the biotin dissociation results were confirmed using ^3^H-biotin (Figure 3E,G). Maleimide had no effect on the ability of wt avidin to bind or release ^3^H-biotin, since the amount of unbound ^3^H-biotin was 2.7±0.1% and the dissociation of the bound ^3^H-biotin 24 hours after the addition of cold biotin was 7%. In contrast, maleimide significantly reduced the ability of Avd(S16C) to bind ^3^H-biotin as the amount of unbound ^3^H-biotin was 25.6±0.1%. Following the addition of a 1000-fold excess of cold biotin, Avd(S16C) rapidly released over 85% of the bound ^3^H-biotin and during 24 hours the release exceeded 90%. Furthermore, Mal-β-ala decreased the ability of Avd(S16C) to bind ^3^H-biotin, since the amount of free ^3^H-biotin was 34.9±0.4% and following the addition of cold biotin the dissociation of the bound ^3^H-biotin reached 100%. With dcAvd-Cys conjugated with maleimide, the amount of unbound ^3^H-biotin was 16.5±0.3% and the addition of cold biotin led to dissociation of 15% of the bound biotin after 24 hours, showing a slight increase compared to wt avidin.

According to both dissociation assays coupling with maleimide increased the dissociation of BF560-biotin and ^3^H-biotin from Avd(S16C), whereas the dissociation from dcAvd-Cys decreased in comparison to non-conjugated proteins. We believe that maleimide blocks most of the S16C-modified binding sites on dcAvd-Cys and the unmodified sites behave like the binding sites of wt avidin. We expect the coupling efficiency of dcAvd-Cys with maleimide to be high, as was in the case of Avd(S16C).

### Dual chain avidin with two different ligands

**Figure 4:**
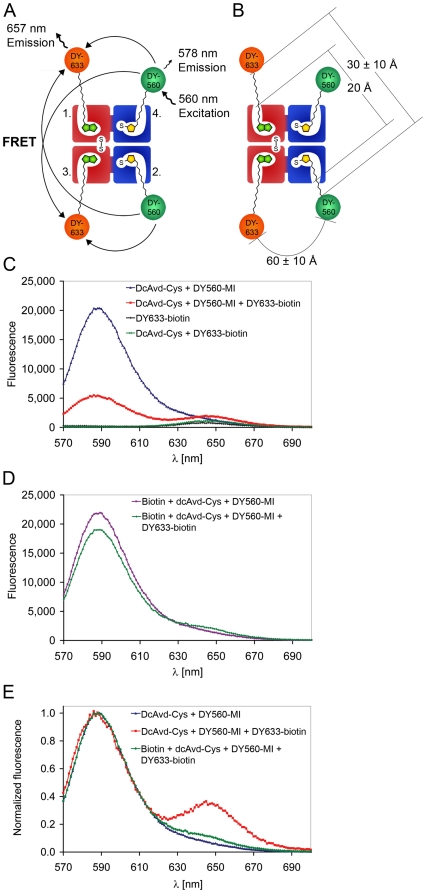
A FRET experiment with dcAvd-Cys. (**A**) A schematic of the FRET of the dcAvd-Cys. The DY560-maleimide (DY560-MI) acting as a FRET donor could be coupled to the Cys16-modified binding sites of dcAvd-Cys, whereas DY633-biotin acting as a FRET acceptor bound to the unmodified binding sites. (**B**) A schematic of the distances of the fluorescent labels attached to dcAvd-Cys. The distance between labels attached to the same side of the dcAvd-Cys molecule is limited by structural features of avidin to 30±10 Å. In contrast, the distance between the labels bound to another cpAvd5→4 - cpAvd6→5 pair over the symmetry axis of the avidin is ∼60±10 Å. (**C, D**) Fluorescence intensity of dcAvd-Cys with a FRET donor and acceptor. The intensities are corrected for sample dilution. (**C**) After exciting the protein complex at 560 nm, the DY560-MI transfers energy to the DY633-biotin, which caused quenching of the donor emission and increased acceptor emission. When the DY633-biotin incubated with dcAvd-Cys or the DY633-biotin label alone was excited at 560 nm the complexes had a minimal fluorescence at 657 nm. (**D**) The quenching of the donor emission decreased when the unmodified binding sites of DcAvd-Cys were blocked with biotin before the addition of DY633-biotin. (**E**) The fluorescence spectra normalized according to the maximum fluorescence at 590 nm of dcAvd-Cys with donor and acceptor present clearly indicated acceptor emission as a consequence of FRET. The acceptor emission was minimal when unmodified binding sites were blocked with biotin before the addition of DY633-biotin indicating that FRET did not occur.

In order to demonstrate the possibility of introducing new molecules to the cpAvd6→5 domain via a maleimide linker we performed a fluorescence resonance energy transfer (FRET) experiment. As a donor we used a fluorescent maleimide (MI) derivative, DY560-MI, which reacts with the introduced thiol group of Cys16 in the cpAvd6→5 domain forming a stable covalent thioether bond. The fluorescent biotin derivative, DY633-biotin acted as the acceptor occupying the unmodified biotin-binding site in the cpAvd5→4 domain. See Figure S2 for structures of DY560-MI and DY633-biotin. After exciting the complex with 560 nm, the DY560-MI was found to transfer energy to the DY633-biotin (Figure 4A). As a result, we saw quenching of the donor emission by 73% and increased acceptor emission (Figure 4C), suggesting the coexistence of both dyes in the same protein tetramers. When the binding sites of the cpAvd5→4 domains were blocked with biotin before addition of DY633-biotin, the emission of the donor was quenched less than 13% (Figure 4D). The fluorescence spectra normalized according to the maximum fluorescence at 590 nm of dcAvd-Cys in the presence of both DY560-MI and DY633-biotin clearly indicated an increase in acceptor emission when the donor and the acceptor were bound to the same protein tetramer (Figure 4E). The increase in acceptor emission was insignificant when the unmodified binding sites were blocked with biotin before the FRET experiment (Figure 4D,E). According to previous studies the fluorescence of another biotin dye (DY630-biotin) increased two-fold after the addition of avidin [19]. Similarly, DY633-biotin also showed increased fluorescence emission accompanied by a slight shift in the emission maximum to the longer wavelength when bound to avidin or to dcAvd-Cys. Still, in the FRET experiments the increase in fluorescence was only about 10% (data not presented), since DY633 was excited at 559 nm, which is the wavelength of the DY560 excitation maximum. This increase alone did not explain the increase of the acceptor emission when both donor and acceptor were present.

The size of the avidin tetramer is about 50×50×40 Å and the distance between neighboring cpAvd5→4 to cpAvd6→5 (subunit one and subunit two, or subunit three and subunit four) biotin tails is roughly 20 Å (Figure 4B) as measured from the avidin 3D-structure (PDB 2AVI) using the VMD 1.8.7 program. The fluorescent labels used in the experiment have ten-atom (DY560-MI) and nine-atom (DY633-biotin) flexible linkers. Therefore, the theoretical maximal distance between labels attached to subunits one and two, or to subunits three and four (for clarification, see also the three-dimensional structure of avidin (Figure 1A), would be ∼50 Å, although the structural features of avidin limit the maximal distance to around (30±10) Å in reality. In contrast, the distance between biotin tails bound to another cpAvd5→4 - cpAvd6→5 pair over the symmetry axis of a dcAvd molecule would be about 30 Å and with linkers (60±10) Å. Taken together, if both DY560-MI and DY630-biotin had been bound to subunits one and two, respectively, or to subunits three and four, FRET should have been very efficient. Therefore, our results suggest the binding of dyes to subunits one and four, or to subunits two and three. This assumption is supported by previous studies showing that the affinity towards biotinylated dyes decreases when more than two dyes are bound per molecule [23], [24]. In other words, binding of DY560 equipped with a short linker might inhibit the binding of DY633-biotin to the neighboring binding site. This phenomenon known as anti-cooperative binding is described elsewhere [24].

The efficiency of bioconjugation was determined as the degree of labeling per tetramer (the measured absorbance data presented as Table S2 in Supporting Information). Labeling of wt avidin yielded no bound DY560-maleimides verifying the specificity of the reaction. The degree of labeling was 0.6±0.1 per dcAvd-Cys pseudotetramer. As a control, Avd(S16C) was also labeled with an identical protocol and the degree of labeling was found to be 0.6 per tetramer. We would have expected a higher bioconjugation efficiency, as results obtained by mass spectrometry suggested nearly complete conjugation of Avd(S16C) both with maleimide and N-ethylmaleimide. Thus the low conjugation efficiency may be associated with the non-optimized structure of linker between maleimide and the dye.

## Discussion

A great number of studies performed during the past two decades have indicated that the biotin-binding pocket of (strept)avidin strongly favors its natural ligand, biotin, and it is thus difficult to develop novel ligands, which are not competed out by biotin [2]. Here we present a novel concept for engineering these biotin-binding proteins. A single point mutation S16C in the ligand-binding site of avidin generates an active thiol group, which can be chemically linked to, for example maleimide, as shown here. As a result, a targeted genetic engineering followed by a mild chemical treatment can now be used to control the activity of avidin. Introducing S16C mutation to dcAvd creates two modified binding sites per pseudotetramer. Both mutant sites can be controlled, while the two remaining binding sites resemble those of wt avidin (Figure 3A). The possibility to further modify both biotin binding sites in dcAvd independently provides new opportunities for creating tailored avidins for applications in bio- and nanotechnology. As demonstrated here, dcAvd-Cys can be used in FRET experiments with maleimide- and biotin-dyes (Figure 4). DcAvd-Cys might also prove to be a valuable building block for creating molecular arrangements, for example on sensor surfaces.

The positioning of Cys16 in the ligand-binding pocket of Avd(S16C) and dcAvd-Cys did not significantly affect protein folding, since the proteins could be produced with standard expression methods and the isolated proteins had normal oligomeric states according to an SDS-PAGE assay. However, replacing a serine residue in the biotin-binding pocket led to a modest decrease in biotin-binding affinity, resembling that of the previously introduced S16A mutation [7]. Importantly, the location of the introduced cysteine residue was selected so that it is not available for disulphide bonding between avidin subunits. This improves the usability of the protein since reducing agents and subsequent laborious buffer exchanges can be avoided.

**Figure 5:**
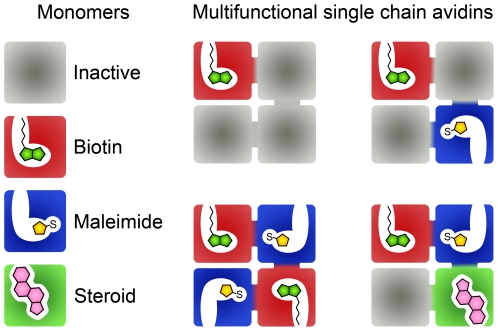
Strategies to selectively modify the binding sites of single chain avidin. Since all four binding sites are encoded by one polypeptide chain, each of them can be modified independently. A combination of the mutations S16R [25] and N118M [15] would lead to a completely inactive monomer, which could be useful in some experiments. In our previous experiments we have generated a steroid binding avidin as an example of an altered binding specificity (Paldanius et al. unpublished results).

The strategy used here could also be applied to the independent modification of binding sites in single chain avidin (scAvd), which has four binding sites in one polypeptide chain [18]. This would make it possible to create avidins with one to four active binding sites per tetramer, freely selecting the positioning of the active sites (Figure 5). The advantage of the Cys-based strategy described here is the wide availability of maleimide reagents, which enable production of tailored proteins to different purposes. In addition, a completely inactive binding site could be created, for example, by combining the mutations S16R [25] and N118M [15]. Furthermore, we have recently modified avidin by random mutagenesis to bind other small ligands. As a proof of principle, avidin was modified to bind testosterone (Paldanius et al. unpublished results). One may also envision an extension to the palette of chemicals used in this scheme. As was demonstrated earlier, Tyr33 in avidin can be specifically modified by tetranitromethane leading to reversible biotin binding [26]. Since it is possible to apply the mutation Y33F to avidin without severe loss in biotin-binding affinity, one could use this to introduce other specific chemical fingerprint to the subunits.

Finally, the properties of covalent and noncovalent binding are different. In the case of avidin, biotin binding can be considered a virtually irreversible reaction. However, if the bond is subjected to mechanical force, the bond lifetime can decrease substantially [27]. Therefore, the technology developed here offer great potential for novel technologies, such as atomic force microscopy (AFM) and molecular recognition force spectroscopy (MRFS), relying on extremely stable and specific molecular bonding.

## Materials and Methods

### Design, production and purification of recombinant proteins

The site-directed mutagenesis of the cDNA encoding avidin, cpAvd6→5 and cpAvd5→4 was performed by the QuikChange (Stratagene, La Jolla, CA, USA) or megaprimer methods [28]. Sequences of primers used in PCR are shown in Table S3. To obtain efficient bacterial secretion the bacterial signal peptide OmpA from *Bordetella avium* was used in front of the Avd(S16C) sequence. The amplified PCR-product was extracted from an agarose gel and subcloned into the pET101/D-TOPO® vector according to the manufacturers instructions (Invitrogen, Carlsbad, CA, USA). Similarly, mutagenized forms of cpAvd5→4(I117C) and cpAvd6→5(S16C, V115H) were joined together by ligation and the complete dcAvd-Cys was subcloned into a pFastBac vector (Invitrogen). All constructs were confirmed by DNA sequencing (ABI PRISM 3100 Genetic Analyzer, Applied Biosystems).

Avidin and Avd(S16C) were produced in *E. coli* BL21-AI cells (Invitrogen) as described in detail earlier [29]. The pET101/D-based expression vectors were transformed into *E. coli* BL21-(AI) cells (Invitrogen). The fresh transformants were cultured in Lysogeny broth (LB) medium with supplements of 0.1% (w/v) glucose and 100 µg/ml ampicillin at 27°C with rotation at 220 rev/min. When culture reached OD_600_ 0.2–0.4 the protein expression was induced by adding 0.2% (w/v) L-arabinose and 1 mM IPTG. Cultivation was continued at 27°C an additional 18 hours before cells were collected by centrifugation (5000 g, 10 min, 4°C).

The Bac-To-Bac® baculovirus expression system (Invitrogen) was used to produce dcAvd-Cys as instructed by the manufacturer and as described in detail previously [22]. Briefly, the pFastBac construct was transformed into *E. coli* DH10Bac cells to generate a recombinant bacmid, which was purified and transfected into *Spodoptera frugiperda* Sf9 insect cells to generate a recombinant baculovirus. The primary baculovirus stock was further amplified for a larger scale production of recombinant dcAvd-Cys. Approximately 2×10^8^ Sf9 cells were seeded to a final volume of 100 ml of HyClone SFX-Insect cell culture medium (Thermo Fisher Scientific, Waltham, MA, USA) without biotin in a 250 ml Erlenmayer flask. Recombinant viruses were added in ratio of 1∶100 and transfected cells were cultured for three days at 28°C with rotation at 125 rev/min. The cells were pelleted by centrifugation (500 g, 10 min, RT).

All produced proteins were purified in a single step with a 2-iminobiotin affinity agarose column (Affiland S. A., Ans-Liege, Belgium). First, cell pellets from *E. coli* cultivations containing avidin and Avd(S16C) were suspended in 30 mM Tris-HCl buffer (pH 8) containing 2 mM EDTA and 20% sucrose. Then lysozyme was added to a final concentration of 2.5 µg/ml and cell lysates were incubated on ice for 30 minutes. After this, 50 mM Tris-HCl buffer (pH 8) containing 2 mM EDTA, 150 mM NaCl and 1% Triton X-100 was added and cells were sonicated two times for five min (50% duty cycle, 5 s on, 3 s off) on ice. This was followed by centrifugation (15 000 g, 30 min, 4°C) of the cell lysate, after which the supernatant was filtered and mixed with an equal volume of binding buffer (50 mM Na-carbonate buffer (pH 11) containing 1 M NaCl). The crude protein mixture was applied to 2-iminobiotin agarose (1 ml), which had previously been equilibrated with binding buffer. The obtained mixture was incubated for one hour on a rolling shaker at 4°C with subsequent centrifugation (500 g, 5 min, RT) and two washing steps with binding buffer. Finally, agarose was transferred to a column and proteins were eluted in one ml fractions with 50 mM sodium acetate buffer (pH 4) containing 100 mM NaCl.

The first steps of purification of dcAvd-Cys from insect cells differed from the steps described above for purifications from bacterial cells. First, the cell pellet from insect cell culture was suspended in 50 mM Tris-HCl (pH 8) buffer containing 2 mM EDTA, 150 mM NaCl and 1% Triton X-100. The obtained cell lysate was sonicated two times for five min (50% duty cycle, 1 s on, 1 s off) on ice. The steps following sonication were similar to those described above for avidin and Avd(S16C) purification.

Following purification all proteins were dialyzed against 50 mM Na-phosphate buffer (pH 7.0) containing 100 mM NaCl. The molecular masses of the proteins were analysed with 15% SDS-PAGE gel stained with Coomassie Brilliant Blue. Proteins were denaturated in SDS-PAGE sample buffer with or without a reducing agent (β-mercaptoethanol) by heating at 95°C for ten minutes. The protein concentrations were determined with a UV/Vis spectrophotometer (NanoDrop 1000 Spectrophotometer, Thermo Scientific, Wilmington, DE, USA) by measuring the absorbance at 280 nm and using an extinction coefficient of 23615 M^−1^cm^−1^ for both avidin and Avd(S16C) monomers and 47355 M^−1^cm^−1^ for the dcAvd-Cys pseudodimer.

### SDS-PAGE-based thermostability assay

Protein samples in the absence and in the presence of D-biotin (Fluka Chemie GmbH, Buchs, Switzerland) were acetylated *in vitro*. An equal volume of SDS-PAGE buffer with β-mercaptoethanol was added and the samples were heated to selected temperatures between RT and 100°C for 20 min. The oligomeric states of the proteins were analysed by 15% SDS-PAGE gel followed by Coomassie Brilliant Blue staining described in detail by Bayer and his coworkers [30].

### Maleimide treatment

Maleimide (Sigma-Aldrich, St. Louis, MO, USA) as well as N-maleoyl-β-alanine (Mal-β-ala, Sigma-Aldrich) were dissolved in DMSO and added in a 100-fold molar excess to the protein solution in 50 mM Na-phosphate buffer (pH 7.0) containing 100 mM NaCl. The samples were incubated at RT for one hour.

### Mass spectrometry

All mass spectrometric measurements were performed with a 4.7-T hybrid quadrupole–FT-ICR instrument (APEX-Qe™; Bruker Daltonics, Billerica, MA, USA), described in detail earlier [31]. Lyophilized avidins were dissolved in HPLC-grade water and desalted with PD-10 columns (Amersham Biosciences, Uppsala, Sweden), equilibrated in advance with 10 mM ammonium acetate buffer (pH 6.8). Desalted protein samples were further concentrated with Microcon (3-kDa cut-off) centrifugal filter devices (Millipore, Billerica, MA, USA). The samples (untreated or treated with maleimide) were further diluted with acetonitrile/water/acetic acid (49.5∶49.5∶1.0, v/v) solution and directly infused into the ESI source at a flow rate of 1.5 µL/min. Maleimide and N-ethylmaleimide (Sigma-Aldrich) were dissolved in HPLC-grade DMSO to a concentration of ∼3 mM, mixed with avidins (ten-fold molar excess of maleimide) and subsequently incubated at RT for 15–30 min before measurements. In-solution trypsin digestion was performed by incubating 150 µl of a 10 µM avidin sample with 0.25 µl of 0.5 mg/ml trypsin (corresponding to ∼1∶40 w/w trypsin∶protein ratio) in 10 mM ammonium acetate buffer (pH 6.8) for four hours at RT. The resulting peptide mixtures were diluted two-fold with acetonitrile/acetic acid (99∶1, vv) and measured immediately. All mass values given in the text refer to the most abundant isotopic masses, either experimentally determined or theoretically calculated from the sequence-derived atomic compositions, unless otherwise stated.

### Biotin dissociation assays

The biotin-binding properties of avidins were studied by dissociation assays using two different methods involving fluorescently labelled biotin (BF560-biotin™, ArcDia Ltd, Turku, Finland) and radioactive biotin ([8,9-^3^H]biotin, PerkinElmer, Waltham, MA, USA). The measurements were performed at RT.

The fluorescence signal from unbound BF560-biotin™ in 3 ml (50 mM Na-phosphate buffer (pH 7.0) containing 650 mM NaCl) was measured for 300 seconds. This was followed by the addition of a two-fold molar excess of protein and the binding of the fluorescent biotin was detected for 300 seconds as quenching of the fluorescence. The fluorescence recovery was measured for one hour after the addition of a 100-fold molar excess of free D-biotin (Fluka Chemie GmbH). A QuantaMaster™ spectrofluorometer (Photon Technology International, Inc., Lawrenceville, NJ, USA) was used to excite the sample at 560 nm and to detect emission at 576 nm. Each measurement was performed twice.

Radioactive ^3^H-biotin at a concentration of 10 nM was incubated overnight with a 50 nM subunit concentration of protein. The unbound ^3^H-biotin was separated from the protein-ligand complex by centrifugal ultrafiltration through 30,000 MW cutoff filters (Vivaspin 500 centrifugal concentrators, Sigma-Aldrich). Measurements were performed in 50 mM Na-phosphate buffer (pH 7.0) containing 100 mM NaCl and 10 µg BSA per ml to prevent non-specific binding. To initiate the dissociation reaction, cold biotin was added to a final concentration of 50 µM and the released ^3^H-biotin was separated from the protein-ligand complex at different time points by ultrafiltration. The radioactivity of the filtrate was analyzed in a Wallac 1410 liquid scintillation counter (Wallac Oy, Turku, Finland). Triplicates of each sample were measured at each time point.

The fraction of bound fluorescent or radioactive biotin at each time point and the dissociation rate constants (k_diss_) were determined using the equation (1):
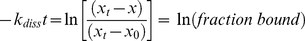
(1)where *x_t_* is the total amount of fluorescent/radioactive ligand before the addition of protein, *x* is the free biotin at each time point and *x_0_* is the amount of free ligand in the presence of protein just before the addition of the competing biotin [11]. The first 500 s were omitted from the data to eliminate the effect of the fast initial-phase characteristic of the avidin-BF650-biotin™ interaction [7].

### Fluorescence resonance energy transfer assay

The FRET donor DY560-maleimide (DY560-MI) (absorption/emission max: 559 nm/578 nm (in ethanol), molar extinction coefficient: 120.000 M^−1^cm^−1^) and the FRET acceptor DY633-biotin (absorption/emission max.: 637 nm/657 nm (in ethanol), molar extinction coefficient: 200.000 M^−1^cm^−1^) for this study were supplied by Dyomics GmbH (Jena, Germany).

Proteins in 50 mM Na-phosphate buffer (pH 7.0) containing 100 mM NaCl were incubated with a five-fold molar excess of DY560-MI for two hours at RT protected from light. The obtained protein-dye conjugate was separated from the free dye by dialysis. The absorption spectrum for the protein-dye conjugate was recorded with a PerkinElmer Lambda 35 UV/Vis spectrometer (PerkinElmer Life and Analytical Sciences) over the full wavelength range. The absorption maxima of the wavelengths 280 nm and 560 nm were used to calculate a dye/protein ratio (5) according to a formula derived from the Lambert-Beer law for the absorption maximum of the dye (2) and for the absorption maximum of the protein at 280 nm adjusted by a correction factor, *CF*, (4) contributed by the dye absorbance at 280 nm. The extinction coefficients used in the calculations were 94460 M^−1^cm^−1^ for the avidin and Avd(S16C) tetramers and 94710 M^−1^cm^−1^ for the dcAvd-Cys pseudotetramer.
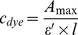
(2)


*ε'  = * molar extinction coefficient of the fluorescent dye
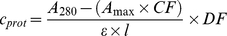
(3)


*DF*, dilution factor. 
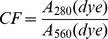
(4)

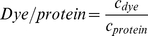
(5)


A QuantaMaster™ spectrofluorometer (Photon Technology International, Inc., Lawrenceville, NJ, USA) was used to excite the sample at 559 nm and to detect the emission spectra from 570 nm to 700 nm. The measurements were performed at RT in a 50 mM Na-phosphate buffer (pH 7.0) containing 100 mM NaCl. First, the emission spectrum was measured for DY560-maleimide conjugated with dcAvd-Cys. This was followed by the addition of DY633-biotin, after which the emission spectrum showing the quenching of the donor emission and the increase of the acceptor emission was measured. As a control measurement the emission spectrum for DY633-biotin alone, and incubated with dcAvd-Cys without DY560-MI were measured. Finally, free biotin was incubated with dcAvd-Cys conjugated with DY560-maleimide and the emission was measured before and after the addition of DY633-biotin. The fluorescence intensities of the measured emission spectra were corrected for sample dilution. Moreover, the fluorescence spectra were normalized according to the maximum fluorescence of dcAvd-Cys in the presence of both DY560-MI and DY633-biotin at 590 nm to clearly indicate the occurrence of FRET when both labels were bound to the same protein tetramer.

## Supporting Information

Figure S1 SDS-PAGE thermal stability analysis of avidin, Avd(S16C) and dcAvd-Cys without (−) and with (+) biotin. The transition temperature was determined after 20 minute heat treatment in the presence of SDS and β-mercaptoethanol followed by SDS-PAGE analysis. M, molecular weight marker.
(TIF)

Figure S2 Structures of fluorescent conjugates used in FRET experiment. (**A**) DY560-maleimide acting as a FRET donor and (**B**) DY633-biotin acting as a FRET acceptor.
(TIF)

Table S1 Trypsin digestion data for Avidin, Avd(S16C) and Avd(S16C) treated with maleimide. Three additional amino acid residues (QTV) originating from the *B. avium* OmpA signal peptide in the N-terminus of the protein are numbered to −3, −2 and −1. Among the identified peptides for Avd(S16C) treated with maleimide, a peptide having a monoisotopic mass of 1947.8630 Da was observed, consistent with the maleimide conjugation into residues 10–26 (highlighted in red). With untreated Avd(S16C), this peptide was absent, but a peptide having a monoisotopic mass of 1850.8512 Da was observed instead, corresponding to the uncoupled tryptic peptide 10–26 (highlighted in red).
(DOC)

Table S2 The degree of labeling per protein tetramer determined by measuring the absorbance at 280 nm and 560 nm for avidin, Avd(S16C), dcAvd-Cys, and for the label, DY560-maleimide (MI).
(DOC)

Table S3 Primers used in polymerase chain reactions (PCR).
(DOC)
