# Supplementary figures and images for: Bifunctional Avidin with Covalently Modifiable Ligand Binding Site
Source: PLoS One. 2011 Jan 27;6(1):e16576. doi: 10.1371/journal.pone.0016576 (PMC3029397; doi:10.1371/journal.pone.0016576)

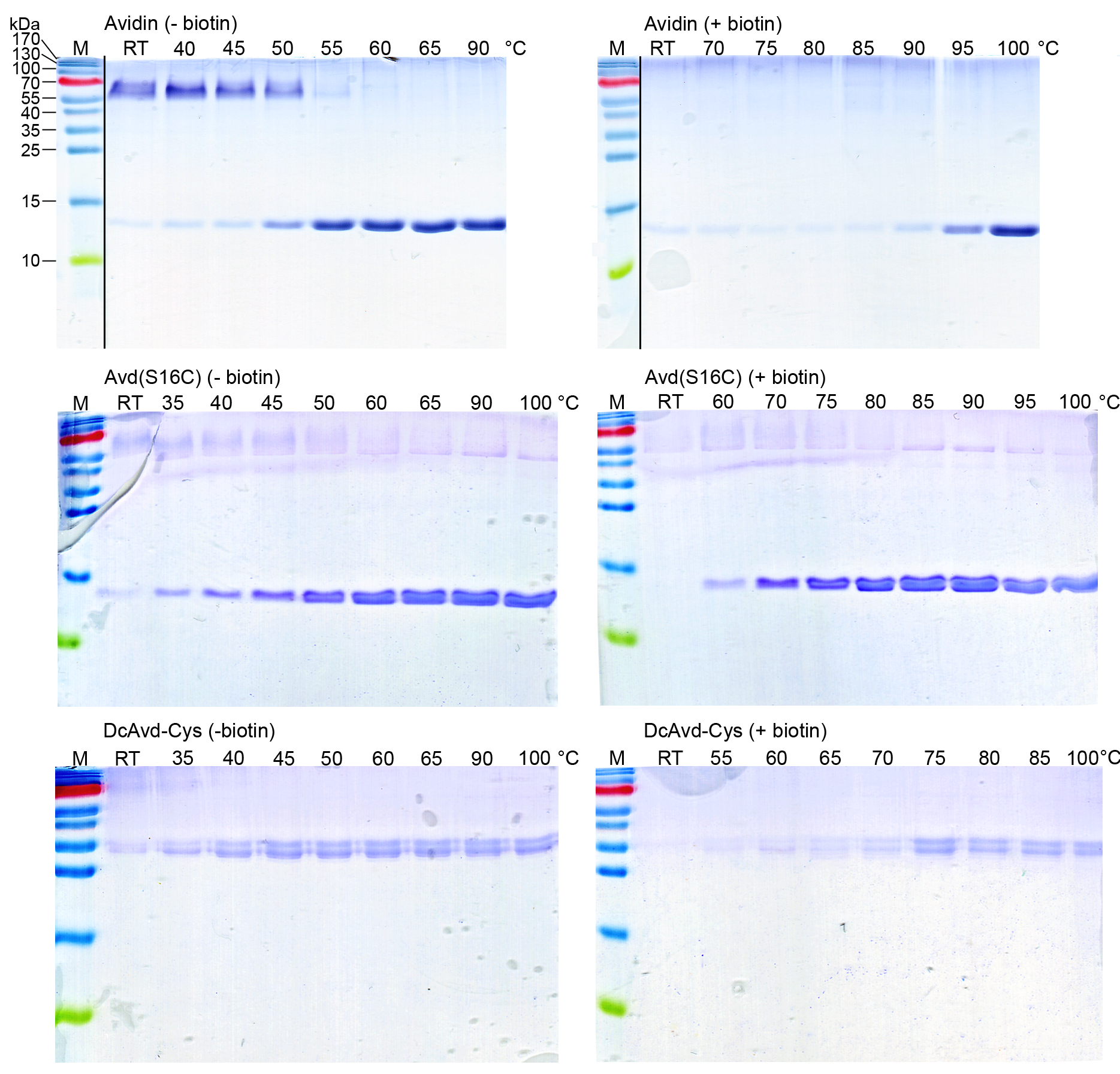

Supplement: Figure S1 — SDS-PAGE thermal stability analysis of avidin, Avd(S16C) and dcAvd-Cys without (−) and with (+) biotin. The transition temperature was determined after 20 minute heat treatment in the presence of SDS and β-mercaptoethanol followed by SDS-PAGE analysis. M, molecular weight marker. (TIF) [file pone.0016576.s001.tif]

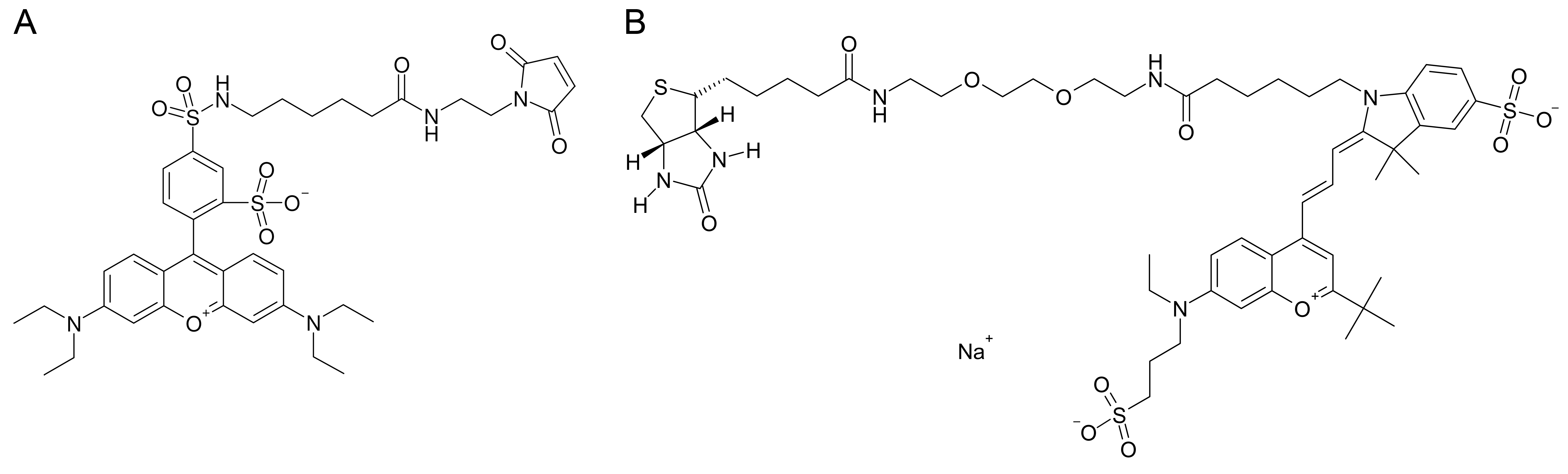

Supplement: Figure S2 — Structures of fluorescent conjugates used in FRET experiment. (A) DY560-maleimide acting as a FRET donor and (B) DY633-biotin acting as a FRET acceptor. (TIF) [file pone.0016576.s002.tif]
