# Supplementary material for: Bifunctional Avidin with Covalently Modifiable Ligand Binding Site
Source: PLoS One. 2011 Jan 27;6(1):e16576. doi: 10.1371/journal.pone.0016576 (PMC3029397; doi:10.1371/journal.pone.0016576)
Supplement: Table S1 — Trypsin digestion data for Avidin, Avd(S16C) and Avd(S16C) treated with maleimide. Three additional amino acid residues (QTV) originating from the B. avium OmpA signal peptide in the N-terminus of the protein are numbered to −3, −2 and −1. Among the identified peptides for Avd(S16C) treated with maleimide, a peptide having a monoisotopic mass of 1947.8630 Da was observed, consistent with the maleimide conjugation into residues 10–26 (highlighted in red). With untreated Avd(S16C), this peptide was absent, but a peptide having a monoisotopic mass of 1850.8512 Da was observed instead, corresponding to the uncoupled tryptic peptide 10–26 (highlighted in red). (DOC) [file pone.0016576.s003.doc]

| **Avidin** |  |  |  |  |
| --- | --- | --- | --- | --- |
| **Peptides identified without modifications:** | | |  |  |
| Experimental (Da) | Theoretical (Da) | Deviation (ppm) | From...to | Sequence |
| 504.2557 | 504.2544 | 2.6 | 125...128 | TQKE |
| 537.3259 | 537.3235 | 4.5 | -3...2 | QTVAR |
| 773.4424 | 773.4395 | 3.7 | 123...128 | LRTQKE |
| 786.4561 | 786.4600 | -5.0 | 88...94 | NGKEVLK |
| 818.4478 | 818.4473 | 0.6 | 95...100 | TMWLLR |
| 918.5328 | 918.5287 | 4.5 | 115...122 | VGINIFTR |
| 1234.5467 | 1234.5466 | 0.1 | 101...111 | SSVNDIGDDWK |
| 1424.7092 | 1424.7088 | 0.3 | 60...71 | TQPTFGFTVNWK |
| 1437.7264 | 1437.7212 | 3.6 | 46...58 | ESPLHGTQNTINK |
| 1562.7338 | 1562.7325 | 0.8 | 101...114 | SSVNDIGDDWKATR |
| 1593.8230 | 1593.8223 | 0.4 | 46...59 | ESPLHGTQNTINKR |
| 1834.8643 | 1834.8632 | 0.6 | 10...26 | WTNDLGSNMTIGAVNSR |
| 2001.9907 | 2001.9895 | 0.6 | 27...45 | GEFTGTYITAVTATSNEIK |
| 3218.6813 | 3218.6796 | 0.5 | 101...128 | SSVNDIGDDWKA...INIFTRLRTQKE |
| 3421.7046 | 3421.7001 | 1.3 | 27...58 | GEFTGTYITAVT...SPLHGTQNTINK |
| 3577.8032 | 3577.8012 | 0.6 | 27...59 | GEFTGTYITAVT...PLHGTQNTINKR |
| 2442.1222 | 2442.1195 | 1.1 | 4...9 + 72...87 | CSLTGK + FSESTTVFTGQCFIDR (Ox) |
| 3210.5698 | 3210.5689 | 0.3 | 4...9 + 72...94 | CSLTGK + FSESTTVFTGQCFIDRNGKEVLK (Ox) |
| 3338.6621 | 3338.6639 | -0.5 | 3...9 + 72...94 | KCSLTGK + FSESTTVFTGQCFIDRNGKEVLK |
| **Peptides identified with modifications:** | | |  |  |
| 556.2972 | 556.2969 | 0.5 | -3...2 | PCA-QTVAR |
| **Sequence coverage 100%** | |  |  |  |

| **Avd(S16C)** |  |  |  |  |
| --- | --- | --- | --- | --- |
| **Peptides identified without modifications:** | | |  |  |
| Experimental (Da) | Theoretical (Da) | Deviation (ppm) | From...to | Sequence |
| 573.3261 | 573.3235 | 4.5 | -3...2 | QTVAR |
| 773.4401 | 773.4395 | 0.8 | 123...128 | LRTQKE |
| 786.4566 | 786.4600 | -4.3 | 88...94 | NGKEVLK |
| 818.4482 | 818.4473 | 1.1 | 95...100 | TMWLLR |
| 918.5299 | 918.5287 | 1.3 | 115...122 | VGINIFTR |
| 1234.5474 | 1234.5466 | 0.6 | 101...111 | SSVNDIGDDWK |
| 1424.7099 | 1424.7088 | 0.8 | 60...71 | TQPTFGFTVNWK |
| 1437.7228 | 1437.7212 | 1.1 | 46...58 | ESPLHGTQNTINK |
| 1562.7349 | 1562.7325 | 1.5 | 101...114 | SSVNDIGDDWKATR |
| 1593.8236 | 1593.8223 | 0.8 | 46...59 | ESPLHGTQNTINKR |
| 1850.8512 | 1850.8403 | 5.9 | 10...26 | WTNDLG**C**NMTIGAVNSR |
| 2001.9918 | 2001.9895 | 1.1 | 27...45 | GEFTGTYITAVTATSNEIK |
| 2438.1157 | 2438.1141 | 0.7 | 4...26 | CSLTGKWTNDLGCNMTIGAVNSR (Ox) |
| 2456.1374 | 2456.1246 | 5.2 | 4...9 + 10...26 | CSLTGK + WTNDLGCNMTIGAVNSR (Ox) |
| 2566.2090 | 2566.2090 | 0.0 | 3...26 | KCSLTGKWTNDLGCNMTIGAVNSR (Ox) |
| 2584.2325 | 2584.2196 | 5.0 | 3...9 + 10...26 | KCSLTGK + WTNDLGCNMTIGAVNSR (Ox) |
| 3218.7015 | 3218.6796 | 6.8 | 101...128 | SSVNDIGDDWKA...INIFTRLRTQKE |
| 3421.7070 | 3421.7001 | 2.0 | 27...58 | GEFTGTYITAVT...SPLHGTQNTINK |
| 3577.8054 | 3577.8054 | 0.0 | 27...59 | GEFTGTYITAVT...PLHGTQNTINKR |
| **Peptides identified with modifications:** | | |  |  |
| 556.2974 | 556.2969 | 0.9 | -3...2 | PCA-QTVAR |
| 684.3928 | 684.3919 | 1.3 | -3...3 | PCA-QTVARK |
| **Sequence coverage 87% (missing residues 72-87, including Cys83)** | | | | |

| **Avd(S16C)+maleimide** | | |  |  |  |
| --- | --- | --- | --- | --- | --- |
| **Peptides identified without modifications:** | | | |  |  |
| Experimental (Da) | | Theoretical (Da) | Deviation (ppm) | From...to | Sequence |
| 773.4404 | | 773.4395 | 1.2 | 123...128 | LRTQKE |
| 818.4484 | | 818.4473 | 1.3 | 95...100 | TMWLLR |
| 918.5302 | | 918.5287 | 1.6 | 115...122 | VGINIFTR |
| 1234.5477 | | 1234.5466 | 0.9 | 101...111 | SSVNDIGDDWK |
| 1424.7103 | | 1424.7088 | 1.1 | 60...71 | TQPTFGFTVNWK |
| 1437.7232 | | 1437.7212 | 1.4 | 46...58 | ESPLHGTQNTINK |
| 1562.7356 | | 1562.7325 | 2.0 | 101...114 | SSVNDIGDDWKATR |
| 1593.8254 | | 1593.8223 | 1.9 | 46...59 | ESPLHGTQNTINKR |
| 2001.9926 | | 2001.9895 | 1.5 | 27...45 | GEFTGTYITAVTATSNEIK |
| 3421.7092 | | 3421.7001 | 2.7 | 27...58 | GEFTGTYITAVT...SPLHGTQNTINK |
| 3577.8068 | | 3577.8012 | 1.6 | 27...59 | GEFTGTYITAVT...PLHGTQNTINKR |
| 3210.5718 | | 3210.5698 | 0.6 | 4...9 + 72...94 | CSLTGK + FSESTTVFTGQCFIDRNGKEVLK (Ox) |
| 3218.6867 | | 3218.6796 | 2.2 | 101...128 | SSVNDIGDDWKA...INIFTRLRTQKE |
| 3338.6648 | | 3338.6639 | 0.3 | 3...9 + 72...94 | KCSLTGK + FSESTTVFTGQCFIDRNGKEVLK (Ox) |
| **Peptides identified with modifications:** | | | |  |  |
| 556.2977 | 556.2969 | | 1.4 | -3...2 | PCA-QTVAR |
| 1947.8630 | 1947.8567 | | 3.2 | 10...26 | WTNDLG**C**NMTIGAVNSR + **maleimide** |
| **Sequence coverage 100%** | | |  |  |  |
