# Supplementary material for: Bifunctional Avidin with Covalently Modifiable Ligand Binding Site
Source: PLoS One. 2011 Jan 27;6(1):e16576. doi: 10.1371/journal.pone.0016576 (PMC3029397; doi:10.1371/journal.pone.0016576)
Supplement: Table S2 — The degree of labeling per protein tetramer determined by measuring the absorbance at 280 nm and 560 nm for avidin, Avd(S16C), dcAvd-Cys, and for the label, DY560-maleimide (MI). (DOC) [file pone.0016576.s004.doc]

| Sample | Avidin | Avd(S16C) | dcAvd-Cys | DY560-MI |
| --- | --- | --- | --- | --- |
| Absorbance at 280 nm | 0.027 | 0.183 | 0.169 | 0.119 |
| Absorbance at 560 nm | 0 | 0.117 | 0.111 | 0.570 |
| Dilution factor | 3 | 1 | 1 | 10 |
| Correction factor |  |  |  | 0.209 |
| ε [M^-1^ cm^-1^] | 94460^a^ | 94460^a^ | 94710^b^ | 120000 |
| c_protein tetramer_ [M] | 8.6 × 10^-7^ | 1.7 × 10^-6^ | 1.5 × 10^-6^ |  |
| c_dye_ [M] | 0 | 9.7 × 10^-7^ | 9.3 × 10^-7^ |  |
| Degree of labeling | 0 | 0.6 | 0.6 |  |

^a^ For tetramer

^b^ For pseudotetramer
