# Supplementary material for: Bifunctional Avidin with Covalently Modifiable Ligand Binding Site
Source: PLoS One. 2011 Jan 27;6(1):e16576. doi: 10.1371/journal.pone.0016576 (PMC3029397; doi:10.1371/journal.pone.0016576)
Supplement: Table S3 — Primers used in polymerase chain reactions (PCR). (DOC) [file pone.0016576.s005.doc]

| Primer | Sequence |
| --- | --- |
| 5’ Avd | 5’-CACCATGAACAAACCCTCCAAATTCGCTCTGC-3’ |
| 3’ Avd | 5’-TTACTCCTTCTGTGTGCG-3’ |
| S16C.1 | 5’-GACCAACGATCTGGGCTGCAACATGACCATCGG-3’ |
| S16C.2 | 5’-CCGATGGTCATGTTGCAGCCCAGATCGTTGGTC-3’ |
